# Body mass index at diagnosis affects disease course in juvenile dermatomyositis

**DOI:** 10.1186/1546-0096-10-S1-A62

**Published:** 2012-07-13

**Authors:** Anjali Patwardhan, Charles H Spencer, Gloria C Higgins, Robert M Rennebohm

**Affiliations:** 1 Nationwide Children's Hospital, Columbus, OH, USA; 2 The Ohio State University and Nationwide Children's Hospital, Columbus, OH, USA; 3 University of Calgary and Alberta Children's Hospital, Calgary, AL, Canada

## Purpose

We investigated the hypothesis that obesity at diagnosis alters the disease course, response to treatment, and complication rate in juvenile dermatomyositis (JDM).

## Methods

Institutional Review Board approval was obtained to retrospectively review the charts of 73 patients with JDM seen in pediatric rheumatology clinic at Nationwide Children’s Hospital over the past 23 years. Data on treatment and outcomes were collected up through the last clinic visit, or July 30, 2010, whichever came first. Body mass index (BMI) was calculated using ‘Centers for Disease Control and Prevention - BMI calculator for children and teenagers’. We used the Wilcoxon two-sample test to compare numerical variables between the group of patients who were obese (BMI >85^th^ percentile) and the group who were non-obese (BMI ≤85^th^ percentile) at their initial visit. We used logistic regression to compare categorical variables between obese and non-obese groups in SAS 9.2.

## Results

Patient age ranged from 0-18 years. Twenty eight patients had age- and sex-specific BMI ≥ 85^th^ percentile, and 45 had < 85^th^ percentile. Average BMI of obese patients was 93^rd^ percentile and of non-obese patients was 46^th^ percentile. Comparatively fewer patients in the obese group received corticosteroid pulses at diagnosis (p=0.027) and developed osteonecrosis (p=0.050). Comparatively more patients in the obese group had elevated muscle enzymes at 12 months (p=0.027), developed calcinosis (p=0.047), received methotrexate for longer time (p=0.040), were treated with plaquenil (p=0.04), continued to take steroids at last follow up (p=0.028), and continued to have active disease at 5 years (p=0.006), 8 years (p=0.050) and 10 years (p=0.045) following diagnosis.

## Conclusion

JDM patients who were obese at diagnosis (BMI > 85^th^ percentile) were less likely to receive pulse corticosteroids and had fewer bone complications. On the other hand, they had a more severe and prolonged disease course. It is not clear if these differences were related treatment choices, or to metabolic differences such as secretion of pro-inflammatory cytokines by adipose tissue, alteration of pharmacokinetics of medications, or altered sensitivity and response of the hypothalamic-pituitary-adrenal axis including response to corticosteroids.

## Disclosure

Anjali Patwardhan: None; Charles H. Spencer: None; Gloria C. Higgins: None; Robert M. Rennebohm: None.

